# Epidemiological surveillance of schistosomiasis outbreak in Corsica (France): Are animal reservoir hosts implicated in local transmission?

**DOI:** 10.1371/journal.pntd.0007543

**Published:** 2019-06-24

**Authors:** Ana Oleaga, Olivier Rey, Bruno Polack, Sébastien Grech-Angelini, Yann Quilichini, Ricardo Pérez-Sánchez, Pascal Boireau, Stephen Mulero, Aimé Brunet, Anne Rognon, Isabelle Vallée, Julien Kincaid-Smith, Jean-François Allienne, Jérôme Boissier

**Affiliations:** 1 Parasitology Laboratory, Institute of Natural Resources and Agrobiology (IRNASA, CSIC). Cordel de Merinas, Salamanca, Spain; 2 IHPE, Univ. Montpellier, CNRS, Ifremer, Univ. Perpignan Via Domitia, Perpignan France; 3 UMR BIPAR, Ecole Nationale Vétérinaire d’Alfort, ANSES, INRA, Université Paris-Est, Maisons-Alfort, France; 4 Groupement Technique Vétérinaire de Corse, Ghisonaccia, France; 5 UMR SPE 6134, CNRS-Université de Corse Campus Grimaldi Bât 018, Université de Corse, Corte, France; 6 UMR BIPAR, Anses, Ecole Nationale Vétérinaire d'Alfort, INRA, University Paris-Est, Animal Health Laboratory, Maisons-Alfort, France; Imperial College London, UNITED KINGDOM

## Abstract

Environmental and anthropogenic changes are expected to promote emergence and spread of pathogens worldwide. Since 2013, human urogenital schistosomiasis is established in Corsica island (France). Schistosomiasis is a parasitic disease affecting both humans and animals. The parasite involved in the Corsican outbreak is a hybrid form between *Schistosoma haematobium*, a human parasite, and *Schistosoma bovis*, a livestock parasite. *S*. *bovis* has been detected in Corsican livestock few decades ago raising the questions whether hybridization occurred in Corsica and if animals could behave as a reservoir for the recently established parasite lineage. The latter hypothesis has huge epidemiological outcomes since the emergence of a zoonotic lineage of schistosomes would be considerably harder to control and eradicate the disease locally and definitively needs to be verified. In this study we combined a sero-epidemiological survey on ruminants and a rodent trapping campaign to check whether schistosomes could shift on vertebrate hosts other than humans. A total of 3,519 domesticated animals (1,147 cattle; 671 goats and 1,701 sheep) from 160 farms established in 14 municipalities were sampled. From these 3,519 screened animals, 17 were found to be serologically positive but were ultimately considered as false positive after complementary analyses. Additionally, our 7-day extensive rodent trapping (i.e. 1,949 traps placed) resulted in the capture of a total of 34 rats (*Rattus rattus*) and 4 mice (*Mus musculus*). Despite the low number of rodents captured, molecular diagnostic tests showed that two of them have been found to be infected by schistosomes. Given the low abundance of rodents and the low parasitic prevalence and intensity among rodents, it is unlikely that neither rats nor ruminants play a significant role in the maintenance of schistosomiasis outbreak in Corsica. Finally, the most likely hypothesis is that local people initially infected in 2013 re-contaminated the river during subsequent summers, however we cannot definitively rule out the possibility of an animal species acting as reservoir host.

## Author summary

There is an increasing interest on the effect of global changes on the transmission of infectious diseases. Both environmental and anthropogenic changes are expected to promote outbreaks and spread of pathogens. In particular, tropical infectious diseases are expected to move towards more temperate latitudes. Until 2013, urogenital schistosomiasis was restricted to tropical and sub-tropical areas. In summer 2013, a schistosomiasis outbreak has emerged in Corsica (France) with more than 100 cases. Corsica is a French Mediterranean island, which is very popular for tourists from throughout Europe due to the natural beauty of the environment. Surprisingly, in summer 2015 and 2016, the contamination has resumed, and schistosomiasis has been classified in the list of French notifiable infectious disease. In this context it has been hypothesised that reservoir vertebrate hosts, either human and/or animal are at the origin of the maintenance of the local transmission. This paper shows that ruminants (cow, sheep and goats) should not play a role of reservoir host but we found that rodents living in the vicinity of the transmission sites have been infected by the parasite. Considering the low abundance of rodents and the low parasitic prevalence/intensity among rodents, it is unlikely that rats play a significant role in the maintenance of schistosomiasis outbreak in Corsica and that other animals or human could maintain the parasite locally.

## Introduction

The increasing movements of humans and animals and the ongoing climate changes at the global scale are expected to promote the emergence or the spread of tropical infectious diseases in temperate areas [1]. In this context, in summer 2013 more than 100 cases of human urogenital schistosomiasis were contracted in a very confined locality in the south of Europe (Corsica, France) [2, 3]. In summer 2014 the incriminated river (i.e. the Cavu) was closed to recreational activities, but during the two next summers, 2015 and 2016, new local contamination events occurred [4, 5]. Molecular analyses performed on parasites from patients infected in 2013 and 2016 revealed that the parasite was the same unique strain [4]. In this scenario, it has been hypothesised that vertebrate hosts, either human, animal or both, would had been the original source and ongoing reservoirs for this parasite strain, maintaining the parasite transmission in this area [4]

Schistosomes are trematode parasites affecting either humans, animals or both, according to the species concerned in tropical and sub-tropical countries [6]. *Schistosoma haematobium* is responsible for human urogenital schistosomiasis, it is widely distributed through sub-Saharan Africa, Egypt, Sudan and the Arabian Peninsula and it is estimated to affect more than 110 million people [7]. *Schistosoma bovis* is a schistosome species infecting livestock (cattle, sheep, goats, pigs, equines, and dromedaries), wild ruminants, and rodents [8]. This parasite species is widely distributed throughout Africa, the Middle East and to a lower extent in the Mediterranean islands and Spain [9]. It is estimated that at least 165 million cattle are infected with schistosomes worldwide causing serious socio-economic damages [10]. Considering the One Health approach, it has recently been proposed to better quantify these economic losses and eventually to treat the schistosomes infected livestock [11].

Surprisingly, the parasite that emerged in Corsica was not a pure *S*. *haematobium* parasite but a hybrid between *S*. *haematobium* and *S*. *bovis* [12]. It has previously been showed that this hybrid was imported from Senegal [2]. *S*. *haematobium* x *S*. *bovis* hybrids have also been reported in Niger, Mali and Benin [13]. Previous studies have found these hybrid parasites in human and rodent hosts [8]. Only two studies have investigated the presence of such hybrid parasites in domestic cattle but did not find any [14, 15]. However, as the own authors recognised, they only searched the blood vessels of the intestinal tract (infection site of pure *S*. *bovis*) and not the vessels of the urinary tract (infection site of pure *S*. *haematobium*), where the hybrids may have passed unnoticed [14, 15]. The fact that *S*. *bovis* was historically known to be present in the Corsican island [16, 17] makes the epidemiological situation much more complex and suggests the likely presence of local reservoir hosts and the potential of local hybridization events. The last study on the presence of *S*. *bovis* dates from 1962 [18] and since that time, *S*. *bovis* has not been investigated. Identifying infected animals in slaughterhouses relies on the detection of worms of approximately 1.5 cm in the cattle mesenteric system. Moreover, clinical manifestations in the *S*. *bovis* infected animals are poorly documented and the disease is mainly sub-clinical and chronic. As a consequence, the presence of *S*. *bovis* in the island is enigmatic and need to be investigated [19].

Another concern is the potential presence of rodent reservoir hosts. It is well known that schistosome parasites have affinity for rodent hosts, as evidenced by the fact that most species of schistosomes can be maintained under laboratory conditions using several rodent species as vertebrate hosts (e.g. mouse, hamster, rat, gerbil or guinea pig). There is experimental evidence that *S*. *bovis* can infect the Nile rat (*Arvicanthis niloticus* [20]) and a wide range of wild rodents has been found to be naturally infected by this parasite [21]: *Arvicanthis niloticus* [8, 20], *Mastomys natalensis* [22], *Praomys albipes*, *Rattus rattus*, *Mastomys coucha* and *Lophuromys flavopunctatus* [23]. However, rodents are poorly compatible for *S*. *haematobium* and only *Mastomys coucha* and *Arvicanthis niloticus* were successfully experimentally infected so far [23]. To our knowledge there is no evidence of natural *S*. *haematobium* rodent infection. Interestingly however, one *S*. *haematobium x S*. *bovis* hybrid parasite were recently detected in *Mastomys huberti* in Senegal [8]. The close genetic similarities between parasites established in Corsica and those present in Senegal hence raise the question whether rodents present in Corsica could constitute potential reservoir hosts locally.

Our aim was to identify if animals (livestock and/or wild rodents) can be reservoir host of zoonotic schistosomes in Corsica. This study included both a large-scale sero-epidemiological survey on ruminants and a rodent trapping campaign in the vicinity of the Cavu where the schistosomiasis transmission is still persisting. We have also experimentally tested the ability of schistosome hybrids from Corsica to infect *Rattus norvegicus* laboratory hosts.

## Methods

### Sero-epidemiological survey of ruminants

#### Sampling area and serum collection

**Fig 1:**
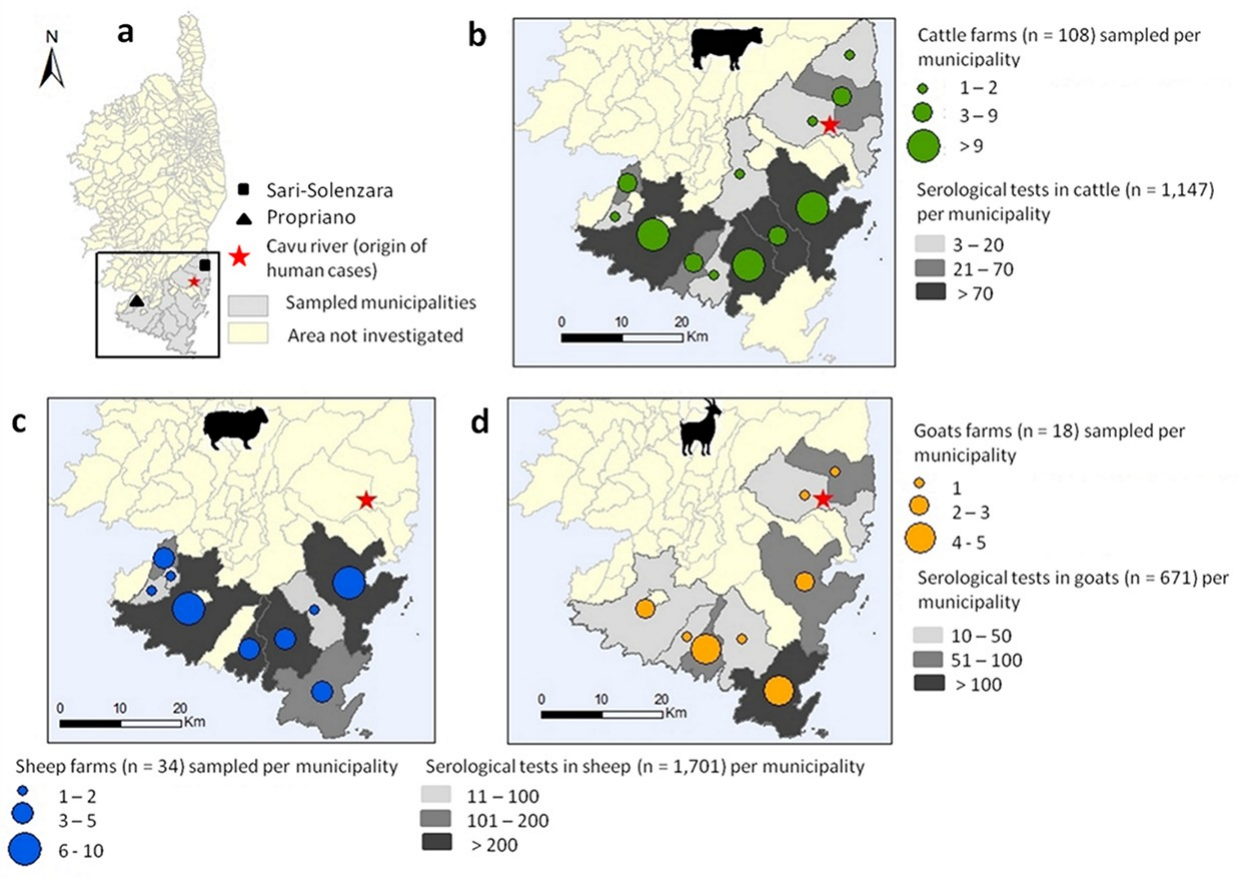
Mapping of the sampling area in south Corsica (a) and distribution of the sampled cows (b), sheep (c) and goats (d). This map was made using QGIS 3.4.1 using open shapefile map of the Corsica island.

Corsica is a French island situated in the north-western Mediterranean Sea at 15 km north from Sardinia and 90 km west from Tuscany in Italy. A large part of the island is preserved as a nature reserve (3500 km^2^), with a mountainous landscape. About 320,000 people live in Corsica. Besides tourism, the local leading economic activity, livestock farming also represents an important resource with approximately 113,000 sheep, 46,000 goats, 55,000 pigs and 65,000 cattle recorded at the island scale [24]. Animals were sampled in an area southwards of a virtual line joining Propriano to Solenzara municipalities that includes (i) the historical *S*. *bovis* foci near the municipalities of Propriano and Sarténe [18] and Monacia-d'Aullène [16] and (ii) the Cavu where human contaminations first occurred in 2013 and 2015 (Fig 1). Blood samples were collected by practicing veterinarians in 2014 and 2015, during the annual serological testing of herds for brucellosis surveillance. In this area, about 16,200 adults' ruminants (7,500 cattle, 7,300 sheep and 1,400 goats) are reared in 255 farms (165 for cattle, 59 for sheep and 31 for goats) localized in 24 municipalities [24].

### ELISA assay

ELISA tests were performed to detect potential anti-schistosome antibodies in the sera of animals using a tegumental extract (TG) of *S*. *bovis* adult worms as coating antigen. This *S*. *bovis* TG extract was obtained as previously described [25].

Multi-well polystyrene plates (Corning, Ref 3369) were coated overnight at 4°C with 0.5 μg/well of TG extract in 100 μl of carbonate buffer, pH 9.6. The following day, the plates were first washed three times with 0.05% Tween-20 in PBS (TPBS) and then blocked with 200 μl/well of 1% bovine serum albumin (BSA) in PBS for 1 h at 37°C. After a series of three additional washes, the sera were placed into duplicated wells (100 μl/well of a 1/100 dilution in TPBS) and incubated for 1 hour at 37°C. After a last washing step, 100 μl/well of a 1/16,000 dilution of peroxidase-labelled anti-bovine IgG (Sigma, A5295) or a 1/6,000 dilution of peroxidase-labelled anti-Sheep/Goat IgG (AbD Serotec, STAR88P) was added and the plates were incubated for 1 hour at 37°C. Finally, the plates were incubated for 10 minutes at room temperature with 100 μl/well of substrate solution (0.6 mg/ml of orto-phenylenediamine and 0.4 μl/ml of H_2_O_2_ in citrate buffer pH 5.0). The reaction was stopped with 100 μl/well of 3N sulfuric acid, and the optical densities (OD) at 492 nm were read with the Multiskan GO spectrophotometer (Thermo Scientific).

In each plate, 3 positive and 3 negative control sera were included. The negative and positive control sera used for the analysis of sheep and goats were obtained from naive sheep and from sheep experimentally infected with *S*. *bovis*. For the analysis of the bovine sera, positive and negative control sera were collected from cattle during an epidemiological study performed in the province of Salamanca (Spain) [26]. Any positive and/or doubtful sera potentially detected were reanalyzed independently following the same protocol. Additionally, bearing in mind that the trematode *Fasciola hepatica* is also present in Corsica [27], the potential reactivity of the TG extract with antibodies against *F*. *hepatica* infection was checked. To do so, serum samples from six lambs experimentally infected with 100 *F*. *hepatica* metacercariae obtained in a previous work [28] were analyzed by ELISA against the excreted/secreted antigen of *F*. *hepatica* (E/S Fh) and the TG extract of *S*. *bovis*. The E/S Fh antigen was prepared as previously described [29].

### Statistics of ELISA data

To analyze the results of the serology and to compare serological patterns between samples, each optical density (OD) was transformed into an Elisa Index (EI) by applying the following formula: OD of each serum / mean OD of the negative control sera included in each plate. Receiver–Operator Characteristic (ROC) curve were built for the TG extract and used to establish the cut-off value of EI used to discriminate between positive and negative animals. A ROC curve is obtained by calculating the sensitivity and specificity of a test or an antigen at every possible cut-off point, and plotting sensitivity against specificity [30]. The current ROC analysis was performed using 50 well-defined sera from sheep infected experimentally with *S*. *bovis* and 164 sera from naive sheep (without schistosomes).After that, the cut-off selected was the EI value that gave the highest diagnostic performance for the TG extract, which was calculated as the sum of the sensitivity and specificity divided by two. Then, this value was used to establish the positive/negative state of all the animal sera analysed. This ROC analysis was performed using the SPSS v17 software package.

### Coprological methods

For ELISA positive animals, coprological examination was assessed by recovering faecal sample directly from the rectum. A faecal sample of approximately 100 grams was diluted in saline solution and passed through a series of stainless steel sieves (420 μm, 250 μm, and 45 μm). The eggs should be retained in the last sieve.

### Rodent trapping

The trapping campaign was done along the Cavu between the 29^th^ of May and the 9^th^ of June 2018. The distance between the river and the traps was generally less than 50 meters and never greater than 150 meters. We have focused our survey in the vicinity of the human transmission sites identified in 2013 and 2015 [2, 4]. The traps (27x9x9 cm; Caussade, France) were deposited along the forest in steep locations and also near human activities. Our protocol considered the nocturnal activity of the targeted species. Thus the traps were baited every late afternoon, and inspected in subsequent early morning. A total of 1,949 traps were placed during 10 trapping sessions. Nontarget species such as shrews (*Crocidura suaveolens*), hedgehogs (*Erinaceus europaeus*) or weasels (*Mustela nivalis*) were immediately released at the point of capture.

Trapped small rodents (mouse and rats) were returned alive to the laboratory for necropsy. Animal were euthanized with lethal injection of 1 mg per kg body weight of a sodium pentobarbital solution (Dolethal, Vetoquinol, Lure, France) and then perfused using an hepatic perfusion technique [31]. The resulting collected blood was filtered to recover potential adult worms. Animal internal organs (i.e. liver, mesenteric vessels, peri-vesical area) were examined to identify possible traces of schistosome infection. Some suspicious zones such as liver cysts and abnormal (hemozoin-like) pigments were biopsied and the fragments of tissues were stored in ethanol 95% for subsequent genetic diagnostics.

DNA extraction from worms and biopsies were performed using E.Z.N.A. tissue DNA extraction kit (Omega Bio-tek, USA) following the manufacturer's instruction. Extracted DNA from worms and biopsies were then amplified by PCR using Sh73/DraI assay [32]. This PCR diagnostic tool is very sensitive and specific to the *S*. *haematobium* parasite group and the resulting genetic profiles allow distinguishing between *S*. *bovis* and *S*. *haematobium* [32]. The PCR target a 121 bp repeated sequence of the schistosome genome. According to the PCR efficiency some extra bands can be observed representing multiples of 121 bp (e.g. 242). For worms only, the complete internal transcribed spacer (ITS) and a partial region of the mitochondrial cytochrome oxidase subunit I (cox1) gene were amplified by PCR and sequenced by a subcontractor (Genoscreen, Lille, France). Sequences were first visualised and manually corrected before being compared to a set of reference sequences from the National Center for Biotechnology Information (NCBI) database including the sequences corresponding to the parasites from the 2013 and 2015 outbreaks [2, 4]. Detailed PCR protocols are available in S1 Text.

### Testing experimental infection of hybrid schistosomes from Corsica on *Rattus norvegicus*

The parasite that emerged in Corsica is routinely maintained in the lab using hamsters as vertebrate hosts and a sympatric lineage of *Bulinus truncatus* as intermediate host [33]. Three Wistar rats (*Rattus norvegicus*) were exposed to three different doses of cercariae: 400, 600 and 800 following standard procedure [31, 34]. Rats were returned to their facilities and reared under standard experimental conditions for 105 day to give time for the potential infecting parasites to develop in their hosts. After this developing period, the rats were euthanized with lethal injection of 0.3 ml of a sodium pentobarbital solution (Dolethal, Vetoquinol, Lure, France)and perfused using hepatic perfusion technique [31]. The blood from each rat was filtered and visually inspected for the presence of adult worms. Similarly to wild animals, organs were also thoroughly examined to identify possible traces of schistosome infection.

### Ethics statement

All experiments were carried out according to national ethical standards (NOR: AGRG1238753A). The French Ministry of Agriculture (Ministère chargé de l’Agriculture), and the French Ministry for Higher Education, Research and Technology (Ministère de l’Education Nationale de la Recherche et de la Technologie) approved the experiments carried out for this study and provided permit A66040 for animal experimentation. The investigator possesses the official certificate for animal experimentation (Decret n˚ 87–848 du 19 Octobre 1987).

## Results

### Sero-epidemiological survey on ruminants

The sera of a total of 3,519 animals from 160 farms localized in 14 municipalities were sampled. This sampling effort represents 15% (n = 1,147), 48% (n = 671) and 22% (n = 1,701) of the total cattle, goats and sheep reared in this area respectively (Fig 1). The resulting sera were stored at -20°C and sent to the laboratory of parasitology at IRNASA (CSIC) in Salamanca (Spain) for ELISA tests.

ROC analysis allowed us to set up a positivity/negativity cut-off between EI 2.5 and 2.8, with a diagnostic performance of 96.7% (97% sensitivity and 99.4% specificity). Since farmed animals are expected to be in contact with a greater number of pathogens and parasites compared to the experimental animals infected under laboratory conditions, we conservatively established a cut-off slightly higher (namely, EI 3). Accordingly, EI values comprised between 2.5 and 3 were considered unclear results and the associated sera were re-analysed. More generally, we applied the following criterion: animals with serum samples displaying EI < 2.5 were considered as negative, those with sera that displayed EI values between 2.5 and 3 were considered as doubtful, and finally animals with a detected EI value > 3 were considered as infected by Schistosomes.

**Fig 2:**
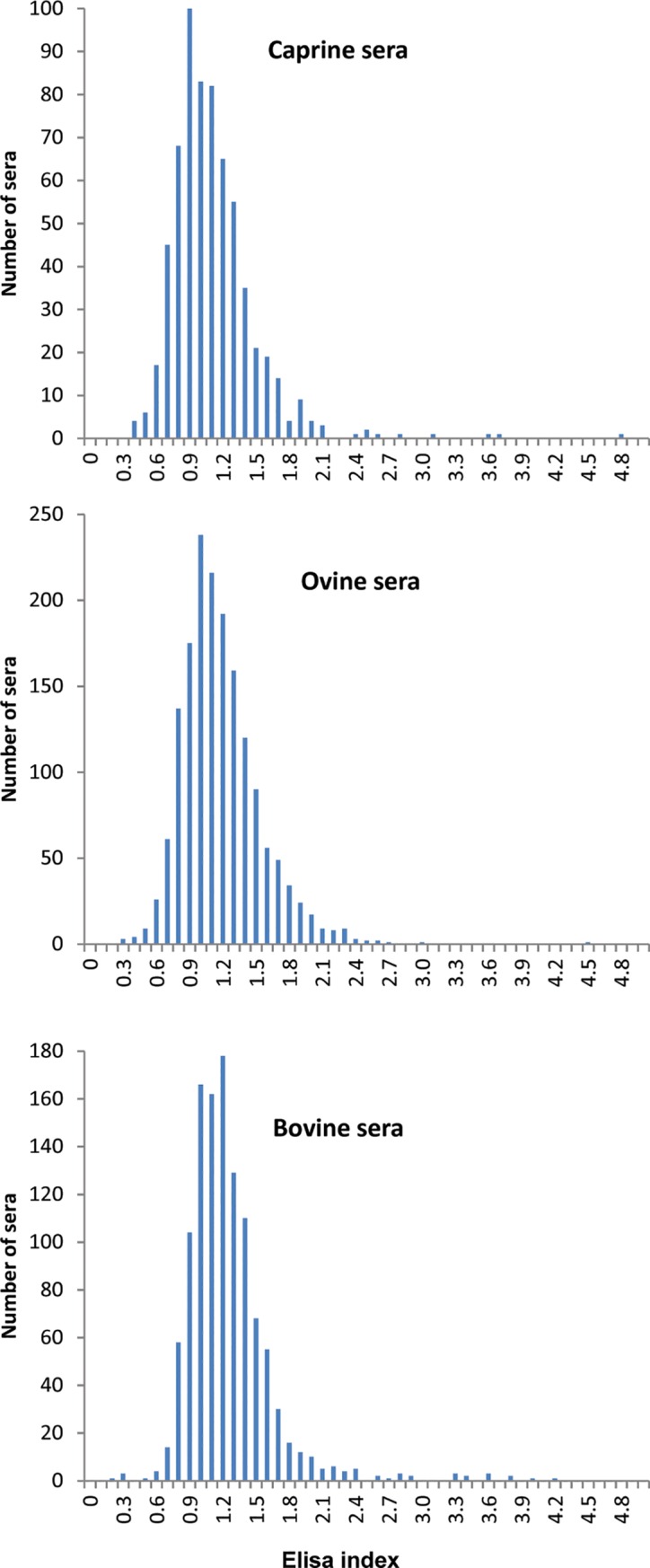
Occurrence of the ELISA index (EI) values given by the analyzed sera samples in goats, cows and sheep.

Table 1 shows the overall results of the ELISA analyses of 3,519 sera and Fig 2 represents the frequency of EI values obtained for each host species (i.e. goat, cattle, sheep). These results indicate that 99% of the sera analysed were negative, 0.52% remained doubtful after a double-analysis and 0.48% were found to be positive with EI values higher than 3. In order to explain these positive reactions, we first analyzed the cross-reaction to *F*. *hepatica*, which is an autochthonous trematode in Corsica [27]. The sera from sheep infected with *F*. *hepatica* showed increasingly detectable levels of antibodies to *F*. *hepatica* from the second week of infection onwards. However, none of these sera reacted at all with the TG antigen, clearly indicating that a possible concurrent infection by *F*. *hepatica* would not interfere in the *S*. *bovis*-ELISA diagnostic test. To confirm the diagnosed positive cases, additional blood samples from some animals found to be seropositive were collected and analyzed independently. From the 17 animals (12 cows– 1 sheep– 4 goats) detected positives during the first serological screening some animals was declared dead by the breeders (1 sheep– 1 goat– 1 cow) and for 8 animals (3 goats and 5 cows), the breeders refused a second serological sampling. Only 6 cows were serologically tested a second time. These cows were also diagnosed based on coprological examinations. This additional series of diagnostic tests revealed that these six cows were in fact negative based on both serological and coprological (i.e. absence of schistosome eggs) examinations.

**Table 1:** Results of the Elisa diagnostic based on the Elisa index (EI) values obtained on individual sera from goats, sheep and cows.

	Negative	Unclear	Positive	
	EI < 2.5	EI = 2.5–3	EI > 3	Total
Goat	663	4	4	671
Sheep	1694	6	1	1701
Cow	1127	8	12	1147

### Rodent trapping

The Table 2 represents the GPS coordinates of the different sites and the number of traps by site. Out of the 1,949 traps placed in the field, a total of 34 rats (*Rattus rattus*), 4 mice (*Mus musculus*),2 shrews (*Crocidura suaveolens*), 3 hedgehogs (*Erinaceus europaeus*) and 2 weasels (*Mustela nivalis*) were trapped.

**Table 2:** Name, GPS coordinates and number of traps for each site.

Site name	Coordinates	Number of traps	Number of trapped animals (*Rattus rattus*)
Les trois piscines*	41°43'56.66"N 9°17'38.11"E	786	6 (5)
Le parc accrobranche*	41°43'26.86"N 9°17'55.09"E	731	24 (15)
Le pont de Conca*	41°42'23.15"N 9°20'0.65"E	298	8 (7)
L'école de Tagliu Rossu	41°42'28.81"N 9°19'3.78"E	80	5 (5)
La prise d'eau	41°42'52.99"N 9°18'14.68"E	54	2 (2)

*: evidence of local human contamination during summer 2013 and 2015.

No trace of schistosome was found in the mice (*Mus musculus*). Among the 34 rats, one individual (called R1) was infected by a unique male schistosome. This rat also displayed granuloma on the liver (that could indicate the past or present occurrence of schistosome eggs in the liver) and black pigments on the mesenteric system. These pigments were similar to the hemozoin pigment specifically found in mature female schistosomes. Both granuloma and a piece of the mesenteric vein harbouring the black pigment were biopsied. A second rat (R2) did not display adult worms after the perfusion but some black pigmentation on the mesenteric system and on the hepatic portal system similar to those found in the rat R1. Biopsies were performed on these two zones. Additionally, samples from the liver, the mesenteric vessels and the bladder from a third rat (R3) where no trace was observed were also biopsied as negative controls.

**Fig 3:**
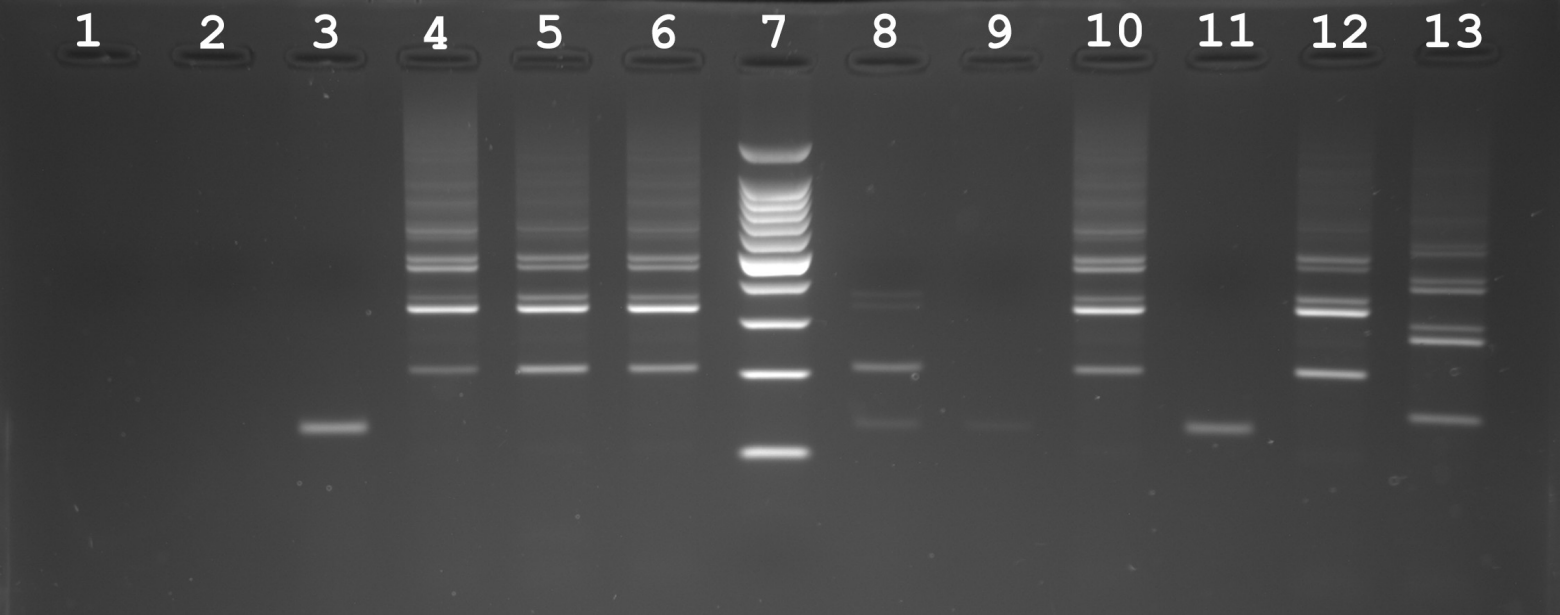
Molecular diagnostic of samples (either worms or biopsies) recovered from non-infected (negative control) and infected rats (*Rattus rattus*) in Corsica, France. The Sh73/DraI PCR specifically targets parasites from the *S*. *haematobium* group. The first three wells correspond to PCR results obtained for biopsies from the liver (well 1), mesenteric vessels (well 2) and bladder (well 3) of a non-infected rat (R3). The following three wells correspond to PCR results obtained from an adult worm (well 4), a mesenteric biopsy (well 5) and a biopsy on a liver granulum (well 6) from the infected rat R1. Well 7 is a size standard (ladder). Well 8–9 are PCR amplifications obtained from a biopsies of the mesenteric vessels (well 8) and of the hepatic portal system (well 9) collected on the infected rat R2. Well 11: negative control. Well 10, 12 and 13 are positive controls (well 10: worm of the corsican schistosome strain, well 12: DNA from pure *S*. *haematobium* worm and well 13: DNA from pure *S*. *bovis* worm). Note that all of the amplification patterns obtained on the infected rats display a classical *S*. *haematobium* pattern.

Fig 3 shows PCR profiles after the Sh73/DraI PCR diagnostic test performed on the biopsies from the two suspicious rats (R1, R2) and the negative non-infected rat R3. No band amplification was observed on the biopsies from the non-infected rat R3 (well 1, 2, 3). Conversely, a PCR amplification was obtained for the adult worm and all biopsies collected on the rat R1 (well 4: worm; well 5: biopsy of mesenters; well 6: biopsy of the granuloma) and all amplifications displayed a migration pattern specific to pure *S*. *haematobium* (well 12). In respect with the rat R2, we find less intense but positive PCR signal (i.e. presence of the 121 bp diagnostic band) on the mesenteric biopsy (well 8). No amplification was obtained on the biopsy performed on the hepatic portal system of the same rat (well 9).

The ITS sequence (Genbank accession number: MK797748) from the single worm from the rat R1 was assigned to *S*. *haematobium* and the cox1 sequence was assigned to *S*. *bovis*, a pattern commonly found in hybrids from the lineage established in Corsica. The cox1 sequence (Genbank accession number: MK797748) obtained from this worm is strictly identical to the Sb2 haplotype found during the 2013 and 2015 outbreaks [2, 4].

### Experimental rodent infection

Among the three Wistar rats experimentally exposed to the schistosome strain that emerged in Corsica only two were slightly infected. No worms were recovered from the rat exposed to 800 cercariae and no trace of schistosomes was observed in the organs. The rat exposed to 600 cercariae displayed one schistosome pair, two single males and a few granulomas on the liver. Finally, the rat exposed to 400 cercariae displayed a single male worm and no granuloma in the liver. No pigment trace was observed on the three animals.

## Discussion

In 2013, the emergence of schistosomiasis in Corsica was clearly unexpected for both the scientific community and public health authorities [35]. Even more surprising was the persistence of the outbreak with evidences of new local infections during summer 2015 and 2016 [4, 36]. Because free larval stages cannot resist in the environment, the persistence of the parasite has to be attributed to the maintenance inside intermediate and/or definitive hosts. Considering the longevity of infected snail intermediate hosts in the lab (170 days at best [37]) and if we suppose that these snails can resist over one winter season this cannot explain the 2015 and 2016 cases. The existence of local vertebrate reservoir hosts either human and/or animal is the more likely explanation.

The diagnostic of schistosomiasis in humans or animals is far from being trivial. For humans, several diagnostic commercial kits exist based on the ELISA, Indirect Haemagglutination (IHA) or Western Blot methods [38]. During the outbreak in Corsica, the French authorities for human health recommended the concurrent use of these two methods (IHA and ELISA), followed by a Western Blot analysis when the two former tests were discordant. For animals, no such commercial kits exist to detect bovine schistosomiasis. Infections of ruminants by *S*. *bovis* can be assessed either directly, i.e. through the detection of parasite eggs in faeces or adult worms in the mesenteric veins in sacrificed animals, or indirectly by detecting antibodies specific to *S*. *bovis* in animals’ blood serum. Coprological diagnosis, which is the most frequently used by veterinarians, has high specificity but is very laborious and offers low sensitivity [39]. The detection of specific antibodies to *S*. *bovis* antigens in the animal host is more sensitive than coprology and furthermore, the serodiagnostic methods (as ELISA) can be applied to large-scale epidemiological studies [40].

In this study, we performed a wide serological survey in domestic ruminants originating from the geographic area where human infections by schistosomes were first reported in south Corsica. We used the *S*. *bovis* TG extract as antigen, which has proved to be very efficient and reliable for the detection of antibodies of *S*. *bovis* in sheep [41]. This method allowed us to detect a total of 17 positive animals. However, for several reasons we believe these positively diagnosed animals were in fact false positives. First, cows found to be serologically positive after the first set of analyses were found to be negative after a second batch ELISA analyses and a sub-sample of these animals were found to be negative using coprological test. Second, *S*. *bovis* usually exhibits a focalized distribution and infected animals are expected to cluster into few particular farms [42]. Contrary to this expectation, the 17 positively diagnosed animals originated from 10 different farms sometimes geographically distant from one another. Third, despite our first technical validation, glycan antigens can be responsible for many cross-reactions between parasite infections [26, 43]. We thus believe that the positive diagnostic of these 17 sera result from unspecific reactions to the glycoproteins that contain the TG extract [41]. In this regard, the potential influence of the hybrid status of schistosomes on the efficiency of serological diagnostic is not known. The sensitivity of serological commercial tests using *S*. *mansoni* antigens is lower for *S*. *haematobium* infection than for *S*. *mansoni* infection [44]. *S*. *bovis* and *S*. *haematobium* are closely related species and *S*. *bovis* antigen has proved to be useful for the detection of human infections by *S*. *mansoni*, *S*. *intercalatum* and also *S*. *haematobium* [45–47]. However, the sensitivity of serological tests needs to be evaluated in the case of hybrid parasite infections in human.

Concerning rodents, both our experimental (i.e. on *R*. *norvegicus*) and field (on *R*. *rattus*) approaches indicate that rats are partially permissive to the hybrid schistosome strain that emerged in Corsica. Unfortunately, we did not experimentally test the compatibility of the schistosome strain naturally present in Corsica to black rats (*R*. *rattus*) since no laboratory strain of *R*. *rattus* was available. Importantly, both the black rats [48–50] and the brown rats [50] are known to be natural alternative reservoir hosts of the parasite *S*. *japonicum*. In the case of *S*. *mansoni* the situation is more complex. The two rodent host species are partially permissive to experimental infections. In particular, adult worms develop in the brown rat but the eggs produced are non fertile eggs and these eggs are not excreted, thus this species is not considered as a suitable vertebrate host for parasite transmission [51]. Conversely, the black rat is known to be a suitable host for *S*. *mansoni* transmission [52]. We lack information concerning the suitability of rats for either *S*. *haematobium* or *S*. *bovis*. Experimental infections showed that both rat species can be infected but are not very permissive to *S*. *haematobium* parasite [53]. To our knowledge only one study showed that *R*. *rattus* is permissive to *S*. *bovis* [23]. The ability of hybrid schistosomes to increase its host range and thus for *S*. *haematobium* hybrids to become a zoonotic parasite is a real concern. To date, no livestock animals was found to be infected by *S*. *haematobium x S*. *bovis* hybrid parasites [14] and only few rodents were found to be infected in Senegal [8]. In the case of Corsica, very few animals were caught (2.3% of trapping success) hence indicating a small abundance of rats in the vicinity of the identified transmission sites. Considering both the low parasite infectivity in experimental infection and the field observation showing low parasite prevalence and intensity in rodents (only two rats infected with very few worms and no trace of reproduction), we suppose that even if rats are permissive to infection their role in the maintenance and transmission of schistosomiasis locally is expected to be negligible.

In summary, our survey strongly suggests that bovine schistosomiasis is absent from the south of Corsica, and livestock or wild rodents may not play a role as reservoir host for human schistosomiasis. The host spectrums of schistosomes are at least partially known in their original distribution area in Africa, Asia and south America, but the invasion of a new ecosystem by a hybrid parasite strain may offer new host possibilities. As an example, the mouflon (*Ovis gmelini musimon*) is endemic from Corsica, and nothing is currently known about the ability of *S*. *bovis* to infect such Ovidae. The fact that *S*. *bovis* is known to infect sheep and the presence of a mouflon population in the Cavu valley could support this last hypothesis. Moreover, the hybrid status of the parasite makes the situation much more complex because the host spectrum of hybrid schistosomes remains enigmatic. Some positive patients emitting schistosome eggs could serve as reservoir for schistosome infection in Corsica. This hypothesis is likely because up to 66% of the patients that have been found to be infected so far were asymptotic [54], the diagnosis is difficult to establish and false negative are possible [54–56], and the median time for first symptom apparition is 30 weeks [54]. To conclude, even if the most likely hypothesis is that local people initially infected in 2013 re-contaminated the river during subsequent summers, we cannot definitively rule out the possibility of an animal species acting as reservoir host.

## Supporting information

S1 Text Primer sequences and PCR protocol. (DOCX)
