# Supplementary material for: Epidemiological surveillance of schistosomiasis outbreak in Corsica (France): Are animal reservoir hosts implicated in local transmission?
Source: PLoS Negl Trop Dis. 2019 Jun 24;13(6):e0007543. doi: 10.1371/journal.pntd.0007543 (PMC6611637; doi:10.1371/journal.pntd.0007543)
Supplement: S1 Text — (DOCX) [file pntd.0007543.s001.docx]

**Primer sequences and PCR protocol**

**DraI/Sh73**

DNA amplifications were performed in a TechneTC-Plus Thermal Cycler. The PCRs were carried out in a total volume of 9 µl using 1 µl of 1/10 diluted DNA template, 0.2mMdNTP, 1.5mMMgCl_2_, 0.32 µM of each primer (see below), 1 U GoTaqG2 (Hotstart, Promega). The PCR conditions were: initial denaturing 3 min at 95°C; 40 cycles of 20 sec at 95°C, 30 sec at 56°C and 10 sec at 72°C; followed by final extension period of 2 min at 72°C. PCR products were visualized on 2.5% agarose gel.

**COI and ITS**

DNA amplifications were performed in a TechneTC-Plus Thermal Cycler. The PCRs were carried out in a total volume of 25 µl using 2 µl of 1/10 diluted DNA template, 0.2mMdNTP, 1.5mMMgCl_2_, 0.32 µM of each primer (see below), 1 U GoTaqG2 (Hotstart, Promega). The PCR conditions were: initial denaturing 3 min at 95°C; 40 cycles of 30 sec at 95°C, 40 sec at 50°C and 1 min 10 sec at 72°C; followed by final extension period of 2 min at 72°C. PCR products were visualized on 2.5% agarose gel. PCR products were sent to Genoscreen (France) for sequencing in both directions using dilutions of the original PCR primers.

| **Sh73/Dra1 primers** | | | |
| --- | --- | --- | --- |
| **Name** |  | **Sequence** | **Reference** |
| 73d | Forward | CCTTGGTCACGTGATTTTC | [1] |
| DraIr | Reverse | TCACAACGATACGACCAAC | [1] |
|  |  |  |  |
| **ITS primers** |  |  |  |
| **Name** |  | **Sequence** | **Reference** |
| ITS4 | Forward | TCCTCCGCTTATTGATATGC | [2] |
| ITS5 | Reverse | GGAAGTAAAAGTCGTAACAAG | [2] |
|  |  |  |  |
| **COI primers** |  |  |  |
| **Name** |  | **Sequence** | **Reference** |
| Cox1_schist_5' | Forward | TCTTTRGATCATAAGCG | [3] |
| Cox1_schist_3' | Reverse | TAATGCATMGGAAAAAAACA | [3] |
|  |  |  |  |

1. Abbasi, I., et al., *Differentiating Schistosoma haematobium from related animal schistosomes by PCR amplifying inter-repeat sequences flanking newly selected repeated sequences.* Am J Trop Med Hyg, 2012. **87**(6): p. 1059-64.

2. Barber, K.E., G.M. Mkoji, and E.S. Loker, *PCR-RFLP analysis of the ITS2 region to identify Schistosoma haematobium and S. bovis from Kenya.* Am J Trop Med Hyg, 2000. **62**(4): p. 434-40.

3. Lockyer, A.E., et al., *The phylogeny of the Schistosomatidae based on three genes with emphasis on the interrelationships of Schistosoma Weinland, 1858.* Parasitology, 2003. **126**(Pt 3): p. 203-24.
